# Triploidy in Citrus Genotypes Improves Leaf Gas Exchange and Antioxidant Recovery From Water Deficit

**DOI:** 10.3389/fpls.2020.615335

**Published:** 2021-02-19

**Authors:** Radia Lourkisti, Yann Froelicher, Stéphane Herbette, Raphael Morillon, Jean Giannettini, Liliane Berti, Jérémie Santini

**Affiliations:** ^1^CNRS, Equipe de Biochimie et Biologie Moléculaire du Végétal, UMR 6134 SPE, Université de Corse, Corsica, France; ^2^CIRAD UMR AGAP, Station INRAE, Corsica, France; ^3^UCA, INRAE, PIAF, Clermont-Ferrand, France; ^4^Equipe SEAPAG, CIRAD, UMR AGAP, F-97170 Petit-Bourg, Guadeloupe, France – AGAP, Univ Montpellier, CIRAD, INRAE, Institut Agro, Montpellier, France

**Keywords:** osmotic adjustment, oxidative status, photosynthesis, polyploidy, water deficit

## Abstract

The triploidy has proved to be a powerful approach breeding programs, especially in *Citrus* since seedlessness is one of the main consumer expectations. Citrus plants face numerous abiotic stresses including water deficit, which negatively impact growth and crop yield. In this study, we evaluated the physiological and biochemical responses to water deficit and recovery capacity of new triploid hybrids, in comparison with diploid hybrids, their parents (“Fortune” mandarin and “Ellendale” tangor) and one clementine tree used as reference. The water deficit significantly decreased the relative water content (RWC) and leaf gas exchange (*P*_*net*_ and *g*_*s*_) and it increased the levels of oxidative markers (H_2_O_2_ and MDA) and antioxidants. Compared to diploid varieties, triploid hybrids limited water loss by osmotic adjustment as reflected by higher RWC, intrinsic water use efficiency (iWUE *P_net_/g_s_*) iWUE and leaf proline levels. These had been associated with an effective thermal dissipation of excess energy (NPQ) and lower oxidative damage. Our results showed that triploidy in citrus enhances the recovery capacity after a water deficit in comparison with diploids due to better carboxylation efficiency, restored water-related parameters and efficient antioxidant system.

## Introduction

Citrus production is one of the main fruit crops worldwide with more than 133 million tons produced in 2017 (FAOSTAT, 2019) and the Mediterranean basin is considered as a secondary area of citrus diversification. Most of the citrus breeding program has focused on the production of seedless fruits (Ollitrault et al., 2008; Aleza et al., 2012; Navarro et al., 2015), the main economic trait for fresh fruit and juice market, with special emphasis in mandarins. Alongside economic issues, citrus production is affected by various environmental constraints. Climate change is expected to induce increased drought episode frequency related to the elevation of average air temperature (Lelieveld et al., 2012) which adversely affect growth, crop yield, and reduce fruit quality, particularly in the arid and semiarid regions around the Mediterranean. Thus, the effective strategy to develop more drought-tolerant varieties is highly relevant to maintain sustainable crop production in the future. The improvement of the citrus fruit market and tolerance to abiotic stresses highlighted the need to develop an efficient strategy to produce seedless citrus varieties withstanding these environmental changes. The modification ploidy level could be an effective way to improve drought stress tolerance in plants and to get seedless fruits.

Polyploidy refers to the presence of more than two sets of chromosomes and offers clear advantages for plant evolution. Polyploidy breeding strategies have been widely exploited for citrus rootstock (Grosser et al., 2015; Tan et al., 2015; Rouiss et al., 2017) and to a lesser extent for citrus scion (Tan et al., 2017, 2019). Citrus trees are mostly diploid (2*n* = 2× = 18), with few exceptions of polyploidy varieties as the triploid “Tahiti” lime. Triploid cultivars are uncommon in the natural environment because of their inviable seeds, resulting in a lack of progeny. However, thanks to their seedlessness and faster growth, triploid plants will be useful for improving phenotypic and organoleptic qualities that are economically important for the fresh fruit market. In citrus cultivars, triploidy is one of the main breeding strategies used to produce innovative seedless fruit (Recupero et al., 2005; Aleza et al., 2012; Navarro et al., 2015) and is mainly achieved by cross-hybridization of diploid × diploid that result from unreduced gametes (Aleza et al., 2016; Rouiss et al., 2017). Triploid production efficiency is determined by the compatibility of parents, pollen viability and the frequency of unreduced gametes, especially of the female parent (Wang et al., 2016). “Fortune” mandarin has been extensively used as a female parent in diploid × diploid hybridization (Aleza et al., 2010; Navarro et al., 2015) because it is more effective for producing a high frequency of triploid hybrids. Triploid plants are heterozygous and often express many desirable characteristics like larger organs, greater vigor and biomass, dark green leaves, and larger organs (fruit and flower) that result in higher yield (Wang et al., 2016). In addition, Jones et al. (2007) reported that triploidy induces an increase in size of somatic and guard cells as well as chloroplasts number, strengthening photosynthesis. Nevertheless, the scarce report on triploidy-induced tolerance (Lourkisti et al., 2020) and the climate changes highlights the need to investigate the resistance properties of triploid plants to abiotic stress, especially water stress. Indeed, improved abiotic stress tolerance has already been reported in polyploids, especially in tetraploids, compared to their diploid counterparts. For example, use of tetraploid citrus has been proved to be an efficient way to enhance tolerance to cold (Oustric et al., 2017), salt stress (Khalid et al., 2020), nutrient deficiency (Oustric et al., 2019), and water deficit (Allario et al., 2013; Dutra de Souza et al., 2017). Nevertheless, most of these studies use tetraploidy for rootstocks breeding while few reports focus on assessing the stress tolerance of economically relevant polyploidy scions.

Water deficit response is a complex phenomenon associated with the maintenance of tissue water contents, stomatal regulation, osmotic adjustment, and antioxidant defense system to limit cell damage (Farooq et al., 2012). Stomatal closure appears to be the main physiological drought-response and aims to minimize water loss through transpiration (Chaves et al., 2003). While a loss of photosynthesis is among the first consequence of stomatal closure (Flexas et al., 1999), some authors suggested that non-stomatal factors (i.e., metabolic processes) become more limiting for photosynthesis, especially under severe water deficit (Lawlor and Cornic, 2002; Chaves et al., 2003). Reduction in CO_2_ uptake can subsequently affect electron transport between photosystems II and I (PSII and PSI) which lead to oxidative stress, through ROS overproduction (Farooq et al., 2009). Although in low doses, ROS (like singlet oxygen, superoxide anion, and hydrogen peroxide) can play beneficial roles in signaling and regulation (Noctor and Foyer, 2016; Foyer et al., 2017), their accumulation is highly toxic and have detrimental effects on proteins, lipids, DNA, and carbohydrates (Mittler, 2002). The risk of irreversible injuries in cells may increase under severe water deficit (Gallé et al., 2007). To prevent damage, plant cells have evolved various drought acclimation and adaptation processes such as antioxidant defenses which could enhance their adaptability to survive during drought stress (Blum, 2017; Wei et al., 2019; Miranda et al., 2020). The antioxidant system is a complex mechanism composed of enzymes (e.g., superoxide dismutase, catalase, ascorbate peroxidase, and dehydroascorbate reductase) and various molecules (ascorbate, glutathione, and proline). Osmotic adjustment is one of the most common response to abiotic stresses including drought, salinity and cold (Serraj and Sinclair, 2002). Osmotic adjustment is referred to the accumulation of solutes like carbohydrates or amino acids and is a major mechanism for drought stress tolerance because it helps in maintaining turgor pressure in the cell while the water potential is decreasing (Pérez-Clemente et al., 2012; Blum, 2017). Proline is one of the most important solute which accumulates in plants subjected to drought stress and it function not only as osmolyte but also as antioxidant, helping ROS detoxification (Szabados and Savouré, 2010; de Campos et al., 2011).

Although the use of triploidy appears to be a successful strategy for dealing with current economic issues, its involvement in improving stress tolerance has yet to be clearly proven. Therefore, the INRAE-CIRAD research centre (San Giuliano, Corsica, France) has developed a citrus breeding program dedicated to triploid citrus hybrids (Ahmed et al., 2020). The hybridization was performed between the “Fortune” mandarin and “Ellendale” tangor used as the female and male parent, respectively, generating population with two ploidy levels (diploid and triploid). The “Fortune” mandarin parent was selected for its pomological traits and its capacity to produce spontaneous triploid genotypes more often than other cultivars. “Ellendale” tangor parent was chosen for its organoleptic quality and its late fruit production (April).

This study aimed to assess the response of triploid citrus varieties to water deficit and their recovery after the stress, an essential step for their future introduction on the market. Their response was compared to diploid varieties, both parents and clementine tree. We analyzed the water status and compared the varieties for the effect of the water deficit treatment on photosynthesis activities through measurements of gas exchange and chlorophyll fluorescence and on the redox status using assays of oxidative markers and antioxidant system.

## Materials and Methods

### Plant Material and Growth Conditions

This study was performed triploid (3×) and diploid (2×) hybrids citrus trees obtained by hybridization between the “Fortune” mandarin (*Citrus reticulata Blanco*) and “Ellendale” tangor (*Citrus reticulata Blanco* × *Citrus sinensis (L) Osb.*). Both 2× parents and 2× common clementine were also selected as reference variety of the study. Diploid and triploid scions were grafted on C-35 Citrange rootstock (*Citrus sinensis “Ruby Blood”* × *Poncirus trifoliata*) and grown in a greenhouse during 7 months at the INRAE-CIRAD experimental station in San Giuliano, Corsica, France (42°17′07.5″ N, 9°31′21.9″ E). C-35 rootstock was chosen for its tolerance to abiotic (drought, cold) and biotic (Tristeza, Phytophthora) stresses. C-35 seedlings used for the experiment were strictly chosen in the nursery to eliminate off-types. The 2× (D1-2× and D40-2×) and 3× varieties (T1-3×, T2-3×, T38-3×, T39-3×, and T40-3×) were selected as described in the previous study (Lourkisti et al., 2020). All plants were transferred in 10-L pots containing a substrate (topsoil, sand, and peat Klasmann TS1, 1:1:2) and grown under controlled conditions (27–30°C day/20–25°C night and the relative humidity ranged from 60 to 80%). The same weight of soil was provided so that the weight of each pot was 7 kg. During the acclimation period, plants were watered near field capacity (FC) and fertilized with a nutritive solution (1 L/h) containing nitrogen, phosphorous, potassium (20-5-10 fertilizer unit), magnesium oxide, and trace elements. This fertilization was supplied according to the recommendations of the local department of agriculture.

### Water Deficit Treatment

One month before the experiment, plants were pruned to homogenize trees in height, architecture and leaf density. To obtain the maximum water holding capacity, the plants were watered until saturation and the excess water was allowed to drain overnight. After draining, the pots were weighed to determine the weight at field capacity (FC). Each pot was then enclosed in a plastic bag to prevent soil evaporation. The experiment was performed in two completely randomized blocks designed as follows: (i) thirty plants (three independent biological replicates per variety) were assigned as control plants and irrigated daily near FC and (ii) thirty others plants (three independent biological replicates per variety) were subjected to water stress so that the soil water content ranged between 35 (threshold at which wilting has been observed) and 45% of FC by manual successive watering. For each pot, when the assigned minimal soil water content was reached, the watering was performed to set the maximal assigned soil water content. To maintain this water condition, each pot was weighed daily before and after irrigation. After maintaining this water stress for 6 days; the physiological parameters were recorded and plant material collected for biochemical analyses. Thereafter, stressed plants were re-watered (recovery period) with daily irrigation near FC for 5 days. Three independent biological leaf and root sample replicates were harvested from each variety and treatment (control, water deficit, and recovery). Leaves and roots samples were immediately frozen and ground in liquid nitrogen and stored at −80°C for biochemical analyses.

### Water Status Parameters

Pre-dawn (Ψ_*PD*_) water potential was determined in one fully expanded leaf per plant (three independent biological replicates per variety), taken from the basal part of the primary branch, using a Scholander-type chamber (PMS Instruments Co., Corvallis, OR, United States). Ψ_*PD*_ was recorded between 04:00 and 06:00 AM before and at sixth day of the water deficit treatment and after the recovery period.

Leaf relative water content (RWC) was determined according to Barrs and Weatherley (1962) between 11:00 AM and 12:00 AM. Three discs from pooled samples of each variety were cut out with a cork borer and immediately weight to determine their fresh weight (FW). The leaf discs were then immersed in distilled water, stored at 4°C in the dark for 24 h and weighed after that to determine their turgid weight (TW). Next, the leaf discs were dried in a forced-air circulation oven at 80°C for 24 h until constant weight and the dry weight (DW) was determined. RWC was calculated according to the following equation:

$$RWC\left(\%\right)=\frac{F\,r\,e\,s\,h\,w\,e\,i\,g\,h\,t-D\,r\,y\,w\,e\,i\,g\,h\,t}{T\,u\,r\,g\,i\,d\,w\,e\,i\,g\,h\,t-D\,r\,y\,w\,e\,i\,g\,h\,t}\times 100$$

### Leaf Gas Exchange and Chlorophyll Fluorescence

Before the beginning of the experiment, five mature leaves per plant were selected and labeled for all physiological measurements (15 independent biological replicates per variety) which were performed between 09:00 AM and 11:00 AM.

Net photosynthesis (*P*_*net*_), stomatal conductance (*g*_*s*_), transpiration (*E*), and intercellular CO_2_ concentration (*Ci*) were monitored with an infrared gas analyzer LCPRO-SD (ADC, BioScientific Ltd., United Kingdom) equipped with the broadleaf chamber at saturating light (PAR at 1400 μmol.m^–2^.s^–1^), at 380 μmol.mol^–1^ for CO_2_ concentration, airflow 200 μmol.s^–1^ while leaf temperature was determined by the environment. Data were recorded when the measured parameters were stabilized (3–6 min). The carboxylation efficiency was estimated as *P*_*net*_/*Ci* while the instantaneous and intrinsic water use efficiencies were calculated as WUE = *P*_*net*_/*E* and iWUE = *P*_*net*_/*g*_*s*_, respectively.

The chlorophyll fluorescence measurements were measured on dark and light-adapted leaves using an OS1p chlorophyll fluorimeter (Opti-Sciences, Inc., Hudson, NH, United States). The application of a saturating actinic light at 3000 μmol photon.m^–2^.s^–1^ was produced by an array of three light-emitting diodes (650 nm) during 1 s and allows us to measure the maximum fluorescence on dark (*F*_*m*_) and light-adapted leaves (*F_m_′*). The maximum(*F*_*v*_/*F*_*m*_) quantum yield of PSII was recorded on dark-adapted (30 min) leaves using leaf clips. In light-adapted leaves, steady-state fluorescence yield was measured and the actual quantum yield of photosystem II was determined as Φ*_PSII_* = *[(F_*m*_′−F)/F_*m*_′]* = Δ*F/F_*m*_*′ (Maxwell and Johnson, 2000). The apparent electron transport rate was automatically estimated by the fluorimeter as ETR = *Φ_*PSII*_* × *PPFD* × *0.5* × *0.84*, where PPFD is photosynthetic photon flux density incident on a leaf, 0.5 is a factor that assumes the equal distribution of energy between the two photosystems, 0.84 represents the leaf absorption (Krall and Edwards, 1992). The ETR/*P*_*net*_ ratio was also calculated to indirectly evaluate the use of electrons by alternative electrons sinks. Non-photochemical quenching (NPQ) was calculated as NPQ = [*(F_m_−F′_m_)/F′_m_*] (Baker et al., 2007).

### Levels of Oxidative Markers

Three independent biological replicates of leaves and roots samples (*n* = 3) for each variety and each treatment were harvested for biochemical analysis.

Malondialdehyde content was determined in leaf and root samples according to Hodges et al. (1999) and adapted to citrus samples as described by Santini et al. (2013). Briefly, 80 mg of sample powder was homogenized in 2 mL 80% ethanol (v/v) and then centrifuged at 3000 × *g* for 10 min at 4°C. Absorbance was determined at 440, 535, and 600 nm against a blank.

Hydrogen peroxide content was assayed using a PeroxiDetect Kit (Sigma, Aldrich, St. Louis, MO, United States) according to Jiang et al. (1991) and adapted to citrus samples as described by Lourkisti et al. (2020). Briefly, 150 mg of sample powder was homogenized in 300 μL of distilled water and centrifuged at 21,000 × *g* for 15 min at 4°C. Absorbance was read at 560 nm with a microplate reader (MULTISKAN FC^TM^, Thermo Scientific, Waltham, MA, United States).

### Levels of Antioxidant Compounds and Enzymatic Activities

Ascorbic acid content was carried out as described by Stevens et al. (2008) and adapted to citrus samples by Lourkisti et al. (2020). Briefly, 150 mg of sample powder was homogenized in 600 μL of 6% ice-cold trichloroacetic acid (w/v) and centrifuged at 13,000 × *g* for 15 min at 4°C. Absorbance was read at 550 nm with a microplate reader (MULTISKAN FC^TM^, Thermo Scientific, Waltham, MA, United States).

Proline content was determined according to Carillo et al. (2011) and adapted to citrus samples by Lourkisti et al. (2020). Briefly, 40 mg of sample powder was homogenized in 70% ethanol (v/v) and centrifuged at 15,000 × *g* for 15 min at 4°C. The absorbance was read at 520 nm with a microplate reader (MULTISKAN FC^TM^, Thermo Scientific, Waltham, MA, United States).

For antioxidant enzymatic activities, sample powder (54 mg) was homogenized in 2 mL of extraction buffer and centrifuged at 13,000 × *g* for 30 min at 4°C. The supernatant was used to determine the SOD, CAT, APX, and DHAR activities as described by Santini et al. (2013). Time-course measurements were read using a V-630 spectrophotometer (Jasco Inc., Tokyo, Japan).

### Statistical Analysis

The data were expressed as means values (±standard error) and analyzed with R statistical software^1^. The qualitative factors studied are sampling period (Control, water deficit, and recovery period) and ploidy level. The variance homogeneity was checked using Levene test and the influence of these factors was analyzed with a two-way ANOVAs followed by the LSD test (*P* < 0.05) when a significant difference was detected. The normality was checked using Shapiro–Wilk test to determine the appropriate correlation coefficient to apply. Pearson’s correlation analysis was performed on normalized data during water deficit conditions and was used to verify the relation between the physiological and biochemical variables under water deficit. Heat map of Pearson’s correlation was performed with RStudio software. Principal Component Analysis (PCA) was performed on normalized data with the FactomineR package bundled with R statistical software. PCA and hierarchical clustering classification (Ward’s method) helps us to better understand the similarity between variables and individuals through the hierarchical distribution of varieties and obtain an optimal number of clusters.

## Results

### Leaf Damages

**FIGURE 1:**
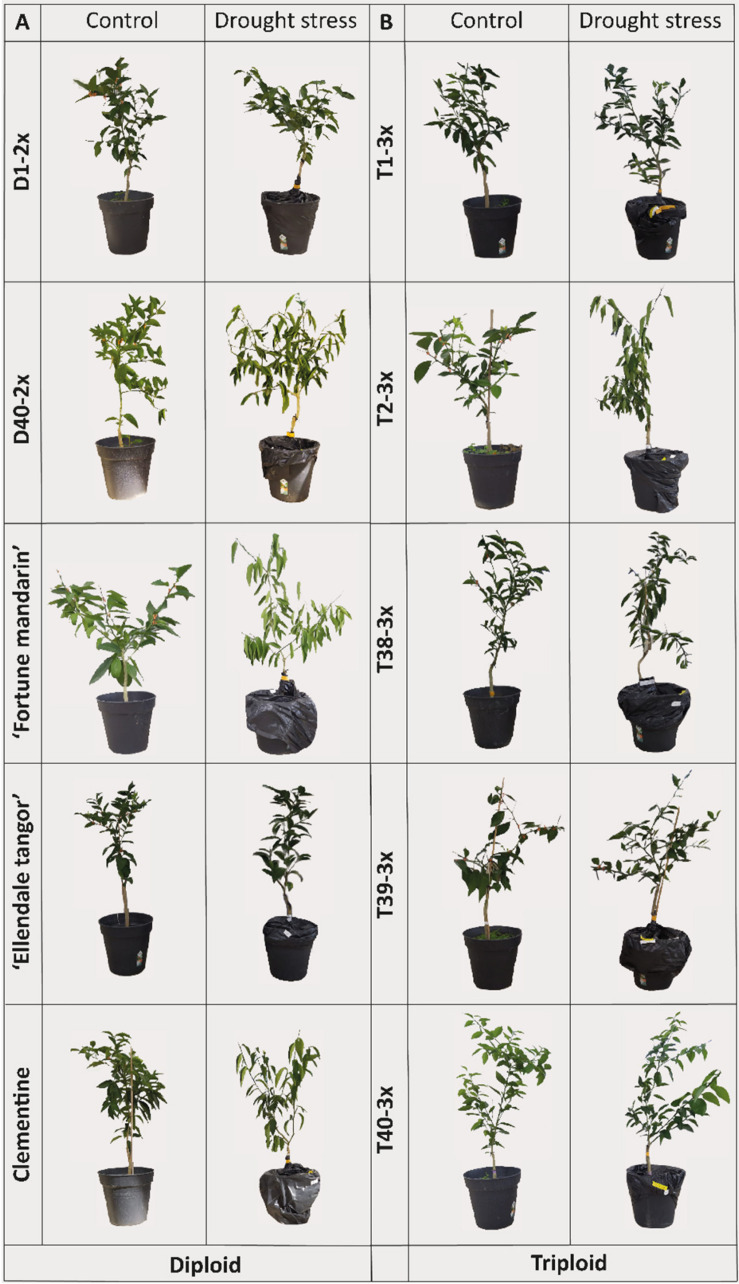
Leaf damages after water deficit (35–45% FC) compared to controls on plants of **(A)** diploid and **(B)** triploid varieties.

Under water deficit conditions, severe leaf wilting and leaf rolling were observed in various 2× varieties (D40-2×, “Fortune” mandarin, clementine) (Figure 1A) while only the T2-3× variety showed similar symptoms (Figure 1B). Additionally, leaf shedding was observed following re-watering in “Fortune” mandarin individuals whereas the T39-3× and T40-3× varieties seems to recover quickly since they developed additional leaves following re-hydration (data not shown).

### Water Status Parameters

**FIGURE 2:**
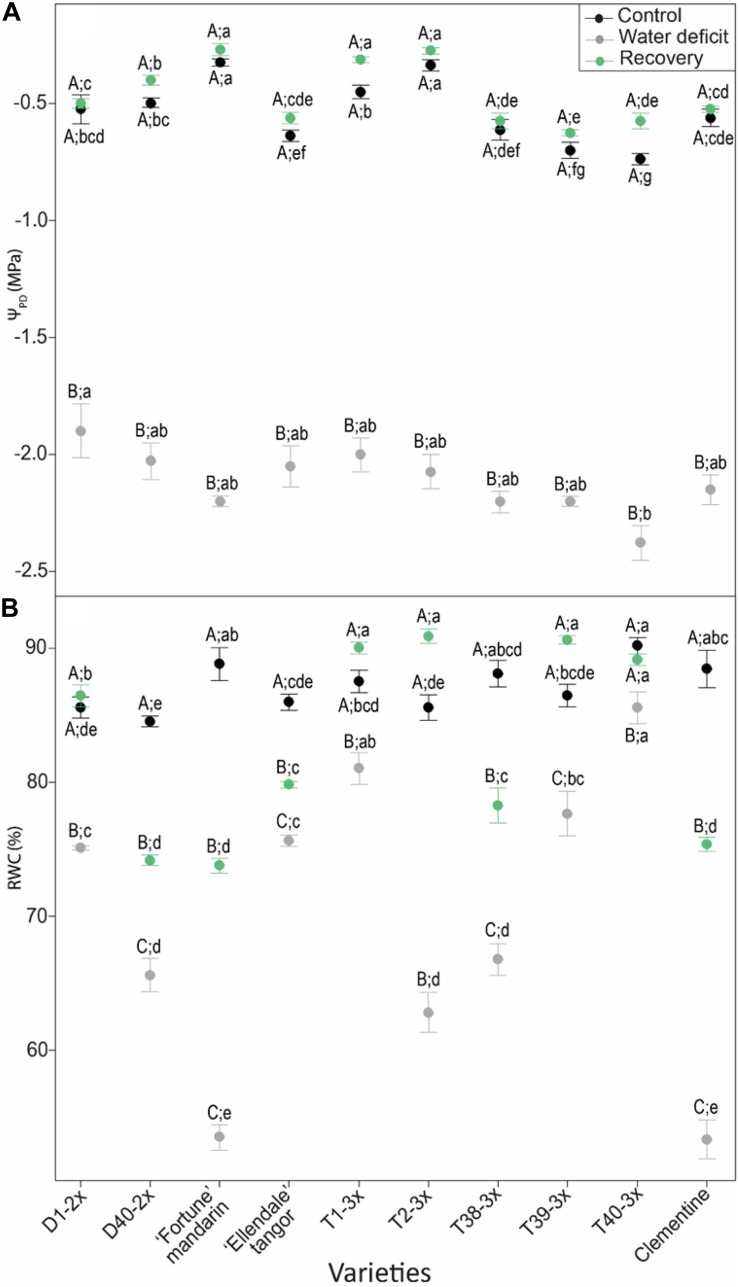
Changes in **(A)** pre-dawn leaf water potential (ΨPD) and **(B)** relative water content (RWC) in varieties under three different water conditions: control (black points), water deficit (gray points), and after rehydration (recovery; green points). All data are mean values (±S.E.) of three independent measurements (*n* = 3). Data were analyzed using ANOVA and Fisher LSD test (*P* < 0.05). Different capital letters indicate significant changes between the conditions (control, water deficit, and recovery) for each variety while different lower case letters indicate changes between the varieties for each condition.

In control plants, pre-dawn leaf water potential (Ψ_*PD*_) was constant through the varieties and remained above −0.7 MPa (Figure 2A). In water stressed plants, Ψ_*PD*_ decreased significantly in all varieties and there was no significant difference between varieties except for D1-2× (−1.90 MPa) and T40-3× (−2.38 MPa). After the recovery period, Ψ_*PD*_ in water-stressed plants increased to reach similar values than those obtained in control plants (Figure 2A). These results showed that watering conditions are similar across varieties, especially for water-stressed plants and allow us to compare the response of the shoot to water stress.

In control plants, the RWC was ranged between 84 and 90% among the varieties (Figure 2B). RWC was significantly affected by water deficit and declined in all varieties. Under these conditions, some 3× varieties (T1-3×, T39-3×, and T40-3×) showed higher RWC than 2× varieties. Additionally, the lowest RWC (54%) was found in 2× varieties (“Fortune” mandarin, clementine) (Figure 2B). After re-watering, RWC increased in all varieties and the highest value was found in 3× varieties (except T38-3×). Despite an increase after the re-watering, RWC of 2× varieties (except for D1-2×) remained lower than the RWC in control plants (Figure 2B).

### Leaf Gas Exchange and Chlorophyll Fluorescence

**FIGURE 3:**
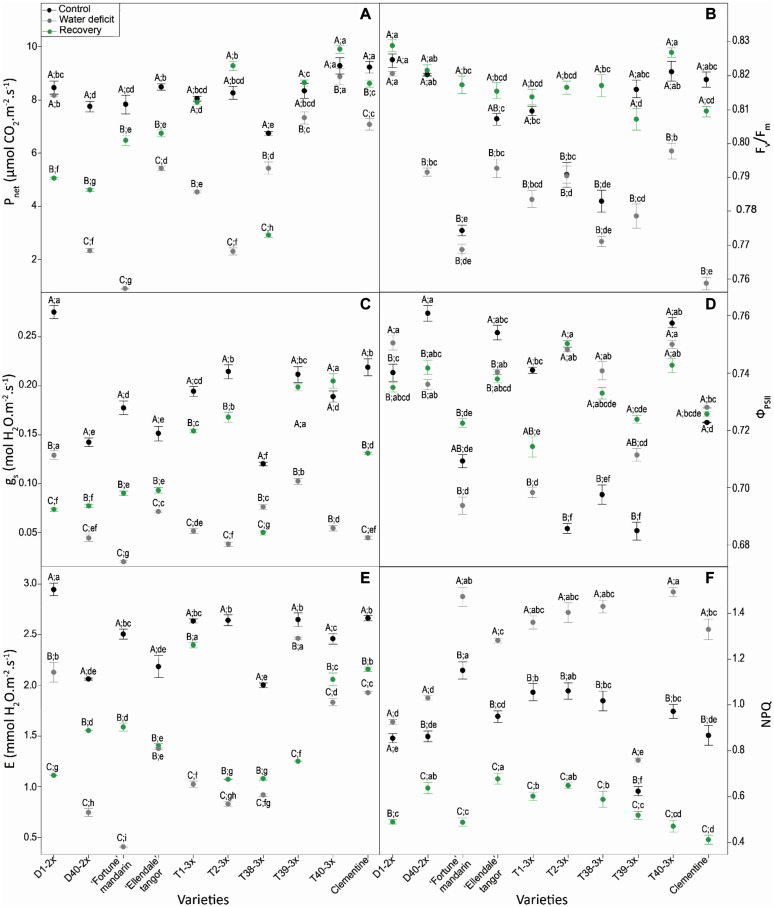
Changes in **(A)** net photosynthesis (*Pnet*), **(B)** maximal quantum yield of PSII (*F*_*v*_/*F*_*m*_), **(C)** stomatal conductance (*gs*), **(D)** actual quantum yield of PSII (Φ_*PSII*_), **(E)** transpiration, and **(F)** non photochemical quenching (NPQ) in citrus varieties under three different water conditions: control (black points), water deficit (gray points) and after re-watering (recovery; green points). All data are mean values (±S.E.) of 15 independent measurements (*n* = 15). Data were analyzed using ANOVA and Fisher LSD test (*P* < 0.05). Different capital letters indicate significant changes between the conditions (control, water deficit, recovery) for each variety while different lower case letters indicate changes between the varieties for each condition.

Net photosynthesis (*P*_*net*_) (Figure 3A), stomatal conductance (*g*_*s*_) (Figure 3C), and transpiration (*E*) (Figure 3E) were significantly affected by water deficit and the responses differed across the varieties. In water-stressed plants, the T40-3× variety (T40-3×) exhibited the highest *P*_*net*_ value while “Fortune” mandarin showed the lowest values of *P*_*net*_, *g*_*s*_, and *E* (Figures 3A,C,E).

The maximum quantum yield of PSII (*F*_*v*_/*F*_*m*_) declined significantly in response to water deficit in all varieties, except for D1-2× (Figure 3B). Under water deficit condition, the weakest *F*_*v*_/*F*_*m*_ value was reported in clementine. After re-watering, *F*_*v*_/*F*_*m*_ fully recovered in all varieties to reach values similar or up to control plants. Differential trends of actual quantum yield of PSII (Φ_*PSII*_) were observed between varieties in response to water deficit (Figure 3D). Overall, the Φ_*PSII*_ values were ranged between 0.75 and 0.70 among the varieties, under water deficit. After the recovery period, few significant differences of Φ_*PSII*_ were found between varieties. NPQ rose significantly in response to water deficit in all varieties (Figure 3F). Overall, most of 3× varieties exhibited greater NPQ values than diploid varieties. “Fortune” mandarin and T40-3× variety exhibited the highest values of NPQ during water deficit. After re-watering, NPQ dropped in all varieties, down to control values.

### Oxidative Status

**FIGURE 4:**
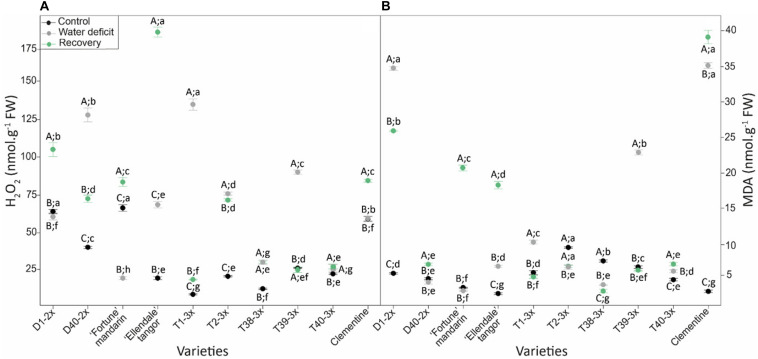
Evolution of **(A)** hydrogen peroxide (H_2_O_2_) and **(B)** malondialdehyde (MDA) contents in leaves of varieties under three different water conditions: control (black points), water deficit (gray points), and after rehydration (recovery; green points). All data are mean values (±S.E.) of three independent measurements (*n* = 3). Data were analyzed using ANOVA and Fisher LSD test (*P* < 0.05). Different capital letters indicate significant changes between the conditions (control, water deficit, and recovery) for each variety while different lower case letters indicate changes between the varieties for each condition.

Oxidative damages in terms of H_2_O_2_ and MDA content were studied in leaves of selected varieties in response to water deficit (Figure 4). H_2_O_2_ and MDA contents were increased or were similar to control values for most of the varieties during the water deficit and during the recovery period (Figure 4). The highest value of H_2_O_2_ content was found in T1-3× while the Fortune mandarin showed the lowest level (Figure 4A). The D1-2× variety and clementine showed the greatest MDA level while the lowest was found in the Fortune mandarin (Figure 4B). After re-watering, a rise in H_2_O_2_ and MDA content was found in most of 2× varieties. The Ellendale tangor and clementine exhibited the highest values of H_2_O_2_ and MDA, respectively. In contrast, the lowest values were found in T38-3× (MDA) and T40-3× (H_2_O_2_) varieties. Overall, 3× varieties fully restored H_2_O_2_ and MDA content close to or below the control values.

**FIGURE 5:**
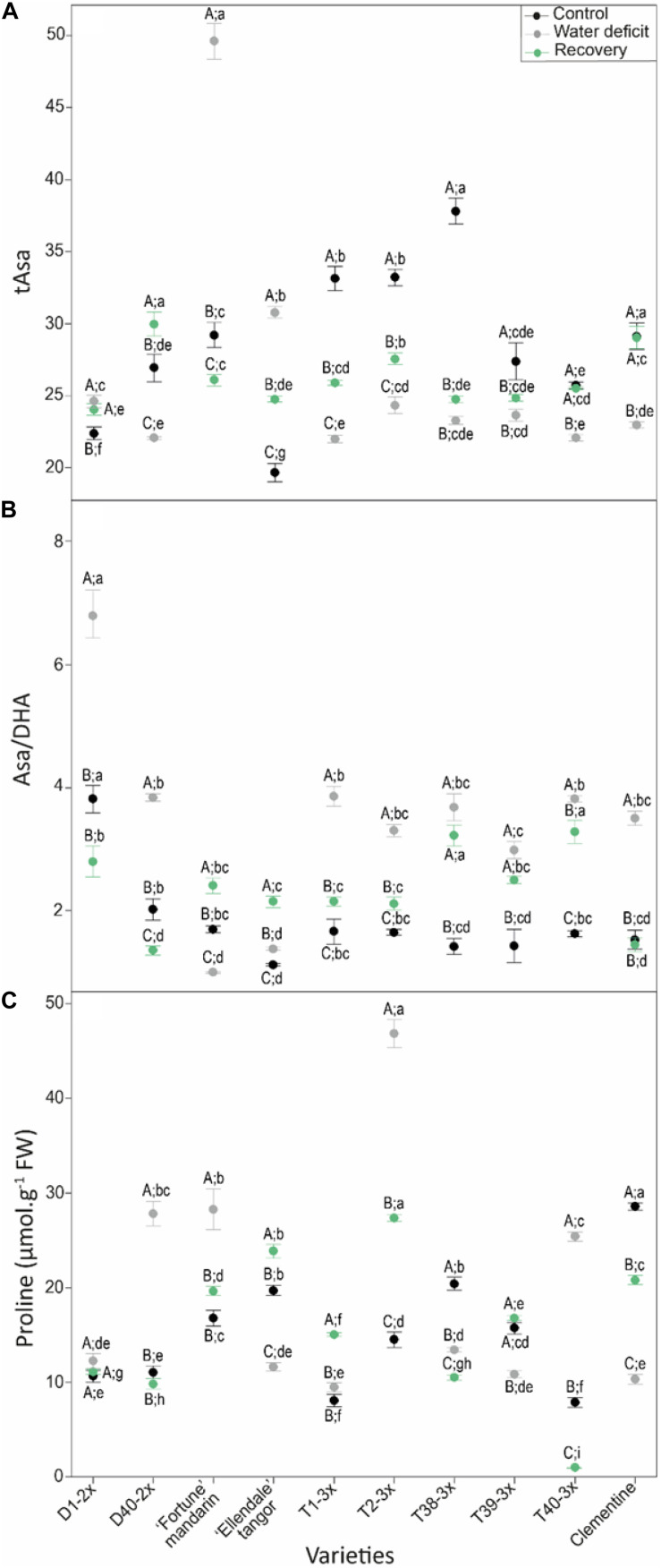
Evolution of **(A)** total ascorbate (tAsa), **(B)** redox status (Asa/DHA), and **(C)** proline contents in leaves of varieties under three different water conditions: control (black points), water deficit (gray points), and after rehydration (recovery; green points). All data are mean values (±S.E.) of three independent measurements (*n* = 3). Data were analyzed using ANOVA and Fisher LSD test (*P* < 0.05). Different capital letters indicate significant changes between the conditions (control, water deficit, and recovery) for each variety while different lower case letters indicate changes between the varieties for each condition.

Antioxidant defenses in terms of tAsa, Asa/DHA ratio and proline were studied in leaves of selected varieties in response to water deficit (Figure 5). Significant changes were reported between 2× and 3× varieties in ascorbate redox status (Figures 5A,B). Overall, high values of tAsa and Asa/DHA ratio were found in the 3× varieties during water deficit and after the recovery period. Different patterns of leaf proline content in response to water deficit and after the recovery period were observed between the varieties (Figure 5C). Most of the 3× varieties, exhibited higher values of proline content under water deficit and after the recovery.

### Pearson Correlation Analysis in 2× and 3× Varieties Under Water Deficit

**FIGURE 6:**
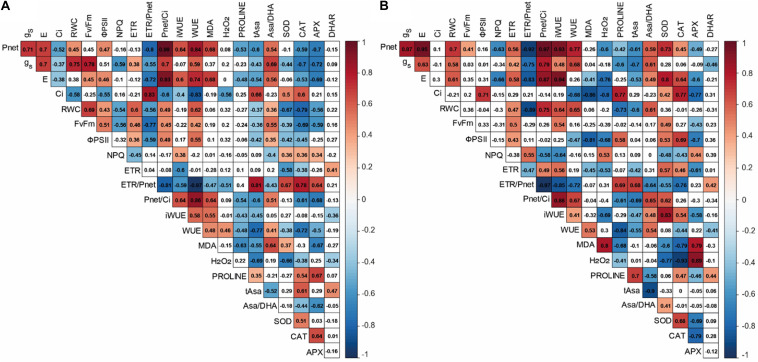
Heat map of correlation matrix of physiological and biochemical variables found in **(A)** diploid and **(B)** triploid varieties under water deficit conditions. Correlation coefficient was calculated based on Pearson’s correlation at significant level (α < 0.05). Red and blue colors indicate positive and negative effects, respectively. Color intensity is proportional to the correlation coefficients, according to the figure legend. The white square corresponds to non-significant correlation (*P*-value > 0.05).

To further examine the water deficit response of 2× and 3× varieties and identify the functional implications and correlations of the various parameters during water deficit, a Pearson correlation analysis was performed (Figure 6). Net photosynthesis was correlated with drought-related parameters (RWC, iWUE, and WUE).

Triploid varieties exhibited negative correlation between NPQ and *P*_*net*_ (*r* = −0.63), E (*r* = −0.66), *P*_*net*_/*Ci* (*r* = −0.58), and iWUE (*r* = −0.64), while NPQ was positively correlated with ETR/*P*_*net*_ (*r* = 0.55) and H_2_O_2_ (*r* = 0.53) (Figure 6B). Proline was negatively correlated with RWC (*r* = −0.73), *P*_*net*_/*Ci* (*r* = −0.61), and WUE (*r* = −0.84) while this osmolyte was positively correlated with ETR/*P*_*net*_ ratio (*r* = 0.69). Hydrogen peroxide (H_2_O_2_) was positively correlated with APX (*r* = 0.89). A positive correlation was also observed between MDA and APX (*r* = 0.79) while this oxidative marker was negatively correlated with SOD (*r* = −0.6) and CAT (*r* = −0.79).

Diploid varieties also exhibited specific correlations under water deficit (Figure 6A). *F*_*v*_/*F*_*m*_ was negatively correlated with ETR/*P*_*net*_ (*r* = −0.77) and positively correlated with *P*_*net*_/*Ci* (*r* = 0.45). A positive correlation was found between ETR/*P*_*net*_ and antioxidant defenses (tAsa, SOD, CAT, and APX).

### Principal Component Analysis for Physiological and Biochemical Responses of Varieties Under Water Deficit and Recovery Period

**FIGURE 7:**
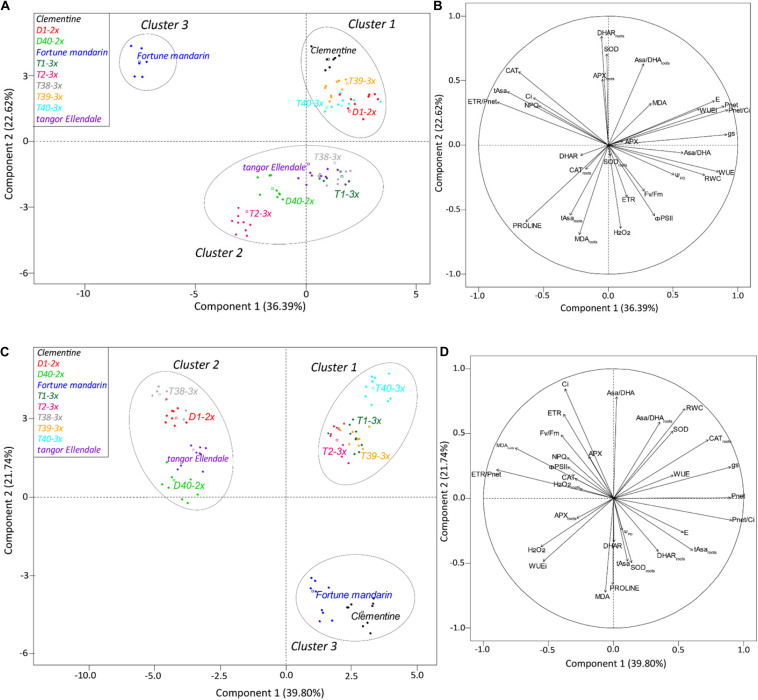
Biplot obtained from PCA performed on leaves of studied citrus varieties under water deficit **(A,B)** and after the recovery period **(C,D)**. Panels **(A–C)** repartition of varieties on the two first axes and **(B–D)** contribution of each physiological and biochemical variable to the two first axes of PCA.

To provide a better understanding of water deficit response of 2× and 3× varieties, physiological and biochemical parameters were submitted to a centered and scaled PCA under water deficit and after the recovery period (Figure 7). The coordinates of the individuals were also analyzed by discriminant analysis to identify a clustering among the varieties based on their response to water deficit (Figures 7A,B) and their recovery capacity (Figures 7C,D). The first two principal components described 59.01 and 61.54% of the total variance in the population under water deficit and after the recovery period, respectively. Under water deficit conditions (Figure 7B), component 1 was positively correlated with gas exchanges related parameters (*P*_*net*_, *g*_*s*_, *P*_*net*_/*Ci*, *E*, WUE, and iWUE) and RWC while it was negatively correlated with some physiological parameters (ETR/*P*_*net*_, NPQ, *Ci*) and antioxidants (tAsa, CAT). Component 2 was positively correlated with leaves (SOD) and roots antioxidants (DHAR_*roots*_, Asa/DHA_*roots*_, and APX_*roots*_) while it was negatively correlated with oxidative status parameters (H_2_O_2_, proline, tAsa_*roots*_, and MDA_*roots*_) and PSII-related parameters (*F*_*v*_/*F*_*m*_, *Φ_*PSII*_*, and ETR). Three main clusters were identified (Figure 7A). Component 1 separated cluster 1 (Clementine, D1-2×, T39-3×, and T40-3×) from cluster 3 (“Fortune” mandarin) while component 2 separated clusters 1 and 3 from cluster 2 (D40-2×, “Ellendale” tangor, T2-3× and T38-3×). Thus, 3× varieties (T39-3×, T40-3×), clementine, and D1-2× variety (cluster 1) were differentiated from the “Fortune” mandarin (cluster 3) by higher photosynthetic performances and better use of water during water deficit. Interestingly, cluster 1 was also characterized by an enhanced antioxidant system in leaves (SOD) and roots (DHAR, Asa/DHA, and APX). The others 3× varieties (T1-3×, T2-3×, and T38-3×), the “Ellendale” tangor and D40-2× variety (cluster 2) were characterized by their enhanced photosynthetic parameters (*F_v_/F_m_*, *Φ_*PSII*_*, and ETR) and accumulation of oxidative markers in leaves (H_2_O_2_) and roots (MDA). They also exhibited the accumulation of antioxidants as proline in their leaves and total ascorbate in their roots.

After the recovery period, the first component was positively correlated with gas exchange parameters (*P*_*net*_, *P_*net*_/Ci*, and *g*_*s*_) and antioxidant molecules in roots (CAT, tAsa) (Figure 7D) while it was negatively correlated with ETR/*P*_*net*_ ratio and MDA accumulation in roots. Component 2 was positively correlated with Asa/DHA ratio, *Ci*, and RWC, whereas it was negatively correlated with antioxidant defenses (proline, DHAR, tAsa, and SOD_*roots*_) and MDA. Component 1 separated cluster 1 (T1-3×, T2-3×, T39-3×, and T40-3×) from cluster 2 (“Ellendale” tangor, D1-2×, D40-2×, and T38-3×). Component 2 separated cluster 3 (“Fortune” mandarin and clementine) from clusters 1 and 2 (Figure 7C). Hence, 3× varieties (cluster 1) were mainly differentiated from 2× varieties (clusters 2 and 3) and T38-3× variety by enhanced photosynthetic capacities (high *P*_*net*_, *g*_*s*_, and carboxylation efficiency *P_*net*_/Ci*) after re-watering. Triploid varieties were also characterized by higher antioxidant defenses in their leaves (Asa/DA ratio and SOD) and roots (CAT, Asa/DHA ratio). “Fortune” mandarin and clementine (cluster 3) were characterized by their increased oxidative markers accumulation (MDA and H_2_O_2_) even if these varieties exhibited enhanced proline and tAsa content in their leaves and higher SOD activity in their roots. Interestingly, the other 2× varieties and T38-3× (cluster 2) were mainly characterized by higher MDA accumulation in their roots and ETR/*P*_*net*_ ratio while physiological traits remained weak after re-hydration.

## Discussion

The decline of water status-related parameters, as the pre-dawn leaf potential (Ψ_*PD*_) and RWC illustrated that the water deficit occurred in all varieties (Figure 2) which negatively affected physiological, and biochemical processes. Poggi et al. (2007) reported that stomatal closure occurs when Ψ_*PD*_ was ranged between −1 and −2 MPa and embolism events start to accumulate in the xylem below −2 MPa. Our conditions are thus constraining enough to induce stomatal closure and thus affected the photosynthetic activity but without inducing a hydraulic failure leading to the plant death. Our conditions also allowed us discriminating varieties for their leaf damages, their physiological and biochemical responses to water deficit illustrating differential performance under these conditions.

### Are Triploid Lines More Tolerant or Resilient to Water Deficit?

Compared to 2× varieties, the 3× varieties appeared less impacted by water deficit (Figure 1). The wilting was reported in 2× varieties, especially D40-2×, the “Fortune” mandarin, and clementine while only one 3× variety (T2-3×) exhibited the same response.

Three main clusters were revealed for the water deficit conditions according to our PCA analysis (Figure 7A). Cluster 1 contained two triploid (T39-3× and T40-3×) and two diploid (D1-2×, clementine) varieties. These varieties appeared to have less disturbance of their photosynthetic performance as indicated by great leaf gas exchange (*P*_*net*_, *g*_*s*_, *E*) and efficient water use, as suggested by high RWC, WUE and iWUE levels (Figures 2B, 7B). Cluster 2 contained three triploid (T1-3×, T2-3×, and T38-3×) and two diploid varieties (“Ellendale” tangor and D40-2×). This cluster exhibited closed water deficit response to cluster 1 in terms of leaf gas exchange and water status parameters, in addition with high *F_v_/F_m_* ratio, Φ_*PSII*_, and ETR values. Taken together, these results suggested a less marked photo-inhibition probably due to a better photo-protection system in both clusters. The “Fortune” mandarin had shown a specific response to water deficit since it is the only variety belonging to the cluster 3. This variety was characterized by a high disturbance of photosynthetic process, higher ETR/*P*_*net*_ values and low levels of carboxylation efficiency (*P_*net*_/Ci*) (Figure 7B). The *P_*net*_/Ci* ratio can be considered as an estimation of Rubisco activity highlighting its limitations under stressing conditions (Niinemets et al., 2009; Silva et al., 2013). Thus, the low levels of *P*_*net*_/*Ci* and iWUE associated with a high ETR/*P*_*net*_ ratio above the physiological value indicated that CO_2_ assimilation could also be limited by biochemical limitations in this variety (Figure 7B and Supplementary Figure 1). Moreover, it has been reported that under water deficit the increased *Ci* values at a very low *g*_*s*_ level reflect impaired photosynthetic metabolism (Flexas et al., 2004). Taken together, these results showed that the “Fortune” mandarin was more sensitive to water deficit than the other varieties.

MDA is one of breakdown product of lipid peroxidation and is used as useful indicator of oxidative damages of plants. It has therefore been used extensively for discriminating citrus cultivars in terms of their tolerance to various environmental stresses (Luna et al., 2000; Tabassum et al., 2017; Lourkisti et al., 2020). In our study, low levels of MDA content were found in 3× varieties, except for T39-3×, indicating that most of 3× varieties were less affected by water deficit-induced oxidative stress, in comparison with 2× varieties (Figure 4B). The less oxidative damages could be due to efficient antioxidant defenses in 3× varieties in response to water deficit. Lower oxidative damages were also observed in tetraploid citrus rootstocks subjected to nutrient deficiency (Oustric et al., 2019) and salinity (Khalid et al., 2020) suggesting an improved tolerance of polyploids citrus to oxidative stresses. ROS such as H_2_O_2_ can also act as secondary messengers for the signaling in ABA-induced stomatal closure (Pei et al., 2000). Merilo et al. (2018) reported that increased H_2_O_2_ content was related to stomatal closure in response to water deficit. In our study, increased H_2_O_2_ content was found in T1-3×, T2-3×, and T39-3× varieties under water deficit (Figure 4A). Associated with high RWC, WUE, and iWUE levels, these results suggested that the better maintenance of water status may be related to an effective H_2_O_2_-related control of stomatal closure (Figure 2B and Supplementary Figure 1). This hypothesis was also supported by the negative correlation between the transpiration rate and H_2_O_2_ found in 3× varieties (Figure 6B). Such trends in sustaining water status have already been reported in polyploidy citrus in response to water deficit (Allario et al., 2013; Oliveira et al., 2017). As indicated by the cluster repartition, we cannot confirm that 3× varieties were clearly more tolerant to water deficit than 2× varieties.

The ability to withstand and to recover from drought is becoming an important issue for tree species as drought episodes are expected to occur more frequently over the next few decades. Our PCA analysis performed after the recovery period, separated clearly the 3× varieties from the 2× varieties according to their recovery capacity. At this period, the three reported clusters were different from those for the water deficit period (Figure 7C). Most of 3× varieties were contained in cluster 1 (except T38-3× variety). These varieties exhibited a better recovery of photosynthetic capacity and water status (Figures 3, 7D). T1-3× and T2-3× varieties clearly exhibited greater recover than “Ellendale” tangor and D40-2× although they belonged to the same cluster under water deficit (Figure 7B). Triploidy would confer a better recovery capacity in terms of gas exchange, oxidative damage and water status parameters, in comparison with diploid plants, following a water deficit. The same conclusions have been reported in triploid *Populus* under water deficit (Liao et al., 2018). Cluster 2 contained diploid varieties (“Ellendale” tangor, D1-2× and D40-2×) and T38-3× variety. These varieties showed high ETR/*P*_*net*_ values, associated with slow leaf gas exchange recovery and carboxylation efficiency, indicating that excessive energy induced by water deficit was not more effectively removed, despite the re-irrigation. The low ability to recover photosynthetic performances was widely associated with drought-sensitive species (Gonçalves et al., 2016; Santana-Vieira et al., 2016; Flexas et al., 2018). The same recovery trends were found between “Fortune” mandarin and clementine, both belonging to cluster 3 (Figure 7C). Despite the relatively high *P*_*net*_ and *E* values of these varieties (Figures 3A,E), the decreased *g*_*s*_ (Figure 3C) and RWC (Figure 2B) suggested that these varieties could not fully recover. The cluster 3 was also characterized by a decreased stomatal conductance related to low chlorophyll fluorescence parameters (*F_v_/F_m_*, Φ_*PSII*_, ETR) and oxidative damage as evidenced by high leaf MDA and H_2_O_2_ content found in the “Fortune” mandarin and clementine (Figure 4). In such conditions, the recovery capacity was closely linked to plant capacity to repair membrane damage during re-watering (Chaves and Oliveira, 2004). In contrast, MDA and H_2_O_2_ accumulation was effectively managed after re-watering in 3× varieties since the values were lower than those found in control plants (Figure 4). We cannot confirm that 3× varieties were clearly more tolerant to water deficit than 2× ones. Nevertheless, triploidy would improve the recovery capacity after a drought episode which may be related to effective photo-protective mechanisms, osmotic adjustment and antioxidant processes.

### Do Photo-Protective Mechanisms, Antioxidants and Osmotic Adjustment Explain the Better Water Deficit Tolerance in 3× Varieties?

Plants have evolved complex photo-protective mechanisms to dissipate excessive energy to limit photo-inhibition. While photochemical mechanisms (photorespiration and Mehler reaction) induced ROS production because of the use of O_2_ as alternative electron sink, NPQ dissipate energy in excess as heat. In our study, water deficit induced excess energy through photosystems in varieties as suggested by the correlation between ETR/*P*_*net*_ and *P*_*net*_/*Ci* (Figure 6). The low decrease in *F_v_/F_m_* (Figure 3B) with the positive correlation between ETR and ETR/*P*_*net*_ found only in 3× varieties (Figure 6B) indicated that the electron flux through the thylakoid membrane is maintained while photosynthesis rate decreased, and excess energy induced by water deficit is effectively dissipated. In addition, the great levels of NPQ associated with the positive correlation between NPQ and ETR/*P*_*net*_ suggested that the thermal dissipation is the main photo-protective mechanism to eliminate the excess energy in 3× varieties (Figures 3F, 6B). Inversely, the photo-protective mechanisms generating ROS production appeared to be less involved in preservation of photosystem apparatus in 3× varieties as illustrated by the negative correlation between ETR/*P*_*net*_ and CAT and SOD (Figure 6B). The involvement of alternative electron sinks in 2× varieties could be evidenced by the negative correlation between *F_v_/F_m_* and enzymes involved in Mehler reaction and photorespiration (SOD, CAT, and APX) (Figure 6A). In response to abiotic stresses, a rise of these alternative electron sinks have already been reported in diploid citrus (Medina et al., 2002; Ribeiro et al., 2009; Machado et al., 2010). Our results showed that both 2× and 3× varieties have set up photo-protective mechanisms to limit water deficit-induced photo-inhibition. While the response to water deficit of the varieties cannot be distinguished by the ploidy level, the 3× varieties exhibited a better water status than 2× varieties during water deficit.

The higher RWC in most of 3× varieties under water deficit (Figure 2B) indicated that they displayed better maintenance of cell homeostasis preventing the water loss (Salehi-Lisar and Bakhshayeshan-Agdam, 2016). Such properties have already been found in polyploidy plants related to drought tolerance mechanisms (Allario et al., 2013; Padoan et al., 2013; Oliveira et al., 2017). It has been shown that *g*_*s*_ decreases faster than *P*_*net*_ at the beginning of prolonged water deficit or during short-term water deficit which causes an increase in the instantaneous water use efficiency (iWUE: *P*_*net*_/*g*_*s*_). In our study, the higher RWC was associated with greater iWUE in 3× varieties (Figure 6B) suggesting the effective prevention of water loss. Osmotic adjustment is a powerful mechanism for preserving cellular water status and proline is the most important compatible soluble (Blum, 2017). Free proline accumulation is a common response of vascular plants to low water potential (Alexieva et al., 2001; Zhu, 2002; Szabados and Savouré, 2010). In our study, the ability of 3× varieties of maintaining the cellular water status during water deficit is supported by the positive correlation between proline with RWC, WUE and *P*_*net*_/*Ci* (Figure 6B). It has been reported that proline accumulation results in enhanced drought tolerance in rice (Dien et al., 2019), wheat (Wang et al., 2019), and polyploidy citrus (Khalid et al., 2020). Moreover, the enhanced proline accumulation in the 3× varieties (Figure 5C) could help in reducing photoinhibition by sustaining electron flow between both photosystems since proline biosynthesis pathway generates NADP^+^ which is the electron acceptor (Cechin et al., 2006). This hypothesis is supported by a ETR/*P*_*net*_ ratio values near to physiological value and by the positive correlation between proline and ETR/*P*_*net*_ found in the 3× varieties under water deficit (Figure 6B). In addition, proline is involved in regulation of cell redox status. de Campos et al. (2011) reported that the great endogenous proline level helps to cope with water deficit in transgenic *Citrumelo* not only by regulating osmotic adjustment but also by helping to alleviate drought-induced oxidative stress. In our study, since few differences in antioxidants enzymes activities were found between the 2× and 3× varieties, the low levels of MDA content found in the 3× varieties (Figure 4B) can be explained by the great proline accumulation and thus low ROS accumulation (Figure 5C). Unlike the other genotypes, T40-3× had lower levels of oxidative markers related to high values of proline and Asa/DHA ratio (Figure 5). Taken together, the osmotic adjustment processes found in 3× varieties may explain the sustained RWC during water deficit and limited photo-inhibition.

After the re-watering, reported cluster containing the 3× varieties (except T38-3×) was characterized by a greater photosynthetic activity, antioxidant system and water status (Figure 7C). The increased SOD activity found in these varieties suggested that effective dismutation of superoxide anion occurred under water deficit and the weaker H_2_O_2_ content observed in their leaves may indicate that H_2_O_2_ detoxification was successfully completed by the CAT and APX, making the plants able to limit oxidative damage (Figure 4 and Supplementary Figure 2). Moreover the high levels of Asa/DHA ratio and enhanced DHAR activity found in 3× varieties (Figure 5B and Supplementary Figure 2) after the re-watering could explained both less oxidative damage and effective protection of PSII since ascorbate act as a co-factor for violaxanthin de-epoxidase in thermal dissipation process (Smirnoff, 2018). Triploidy would confer an improved recovery performance, in comparison with diploid plants, following a water deficit. The same conclusions have been reported in triploid *Populus* under water deficit (Liao et al., 2018).

Overall, our results suggest that triploid varieties can withstand water deficit, preventing photoinhibition and limiting oxidative damages; however ploidy level did not discriminate water deficit response of varieties. Nevertheless, the clear difference in recovery patterns between the 3× and the 2× varieties can be mainly explained by the efficient network of photoprotective mechanisms; the enhanced capacity of osmotic adjustment and the effective antioxidant processes suggested that triploidy improves the resilience capacity after a water deficit episode.

## Conclusion

In this study, we found that triploid varieties were not more tolerant than 2× varieties to water deficit but were clearly distinguished by their improved recovery capacity. Although the antioxidant system does not differentiate 2× from 3× behavior, some 3× varieties appeared to accumulate more proline under water deficit alleviating ROS accumulation and allowing an effective osmotic adjustment. Triploidy clearly increases adaptability to recover photosynthetic and biochemical processes after re-watering can be explained by the greater carboxylation efficiency, restored water-related parameters, greater antioxidant processes and low oxidative damages. Since water scarcity impose significant reduction in crops yield and quality production, plant recovery capacity after one or several successive drought episodes represents a key factor for crops sustainability. Thus, triploidy may offer a powerful tool for the citrus breeding program generating varieties which can withstand and survive under increased frequency and intensity water deficit episode suggested by the scenarios of global environment change. Hence, the response of 3× varieties to several drought episodes under field conditions, associated with impact on fruit growth, should be the focus of future research. Additionally, as the 3× varieties represent an economic interest, the impact of drought stress on fruit yield and quality could also be relevant to provide valuable information for crop improvement.

## Data Availability Statement

The original contributions presented in the study are included in the article/Supplementary Material, further inquiries can be directed to the corresponding author/s.

## Author Contributions

RL collected test data, performed the statistical analyses, interpreted the results, and drafted the manuscript. JS interpreted the results and drafted the manuscript. LB, JG, SH, and RM helped draft the manuscript. All authors contributed to the article and approved the submitted version.

## Conflict of Interest

The authors declare that the research was conducted in the absence of any commercial or financial relationships that could be construed as a potential conflict of interest.

## Footnotes

1 http://www.R-project.org

## References

[B1] Ahmed D. Evrard J.-C. Ollitrault P. Froelicher Y. (2020). The effect of cross direction and ploidy level on phenotypic variation of reciprocal diploid and triploid mandarin hybrids. *Tree Genet. Genom.* 16:25. 10.1007/s11295-020-1417-7

[B2] Alexieva V. Sergiev I. Mapelli S. Karanov E. (2001). The effect of drought and ultraviolet radiation on growth and stress markers in pea and wheat. *Plant Cell Environ.* 24 1337–1344. 10.1046/j.1365-3040.2001.00778.x

[B3] Aleza P. Cuenca J. Juárez J. Navarro L. Ollitrault P. (2016). Inheritance in doubled-diploid clementine and comparative study with SDR unreduced gametes of diploid clementine. *Plant Cell Rep.* 35 1573–1586. 10.1007/s00299-016-1972-4 27038940

[B4] Aleza P. Juárez J. Hernández M. Ollitrault P. Navarro L. (2012). Implementation of extensive citrus triploid breeding programs based on 4x × 2x sexual hybridisations. *Tree Genet. Genom.* 8 1293–1306. 10.1007/s11295-012-0515-6

[B5] Aleza P. Juárez J. Ollitrault P. Navarro L. (2010). Polyembryony in non-apomictic citrus genotypes. *Ann. Bot.* 106 533–545. 10.1093/aob/mcq148 20675656 PMC2944972

[B6] Allario T. Brumos J. Colmenero-Flores J. M. Iglesias D. J. Pina J. A. Navarro L. (2013). Tetraploid Rangpur lime rootstock increases drought tolerance via enhanced constitutive root abscisic acid production. *Plant Cell Environ.* 36 856–868. 10.1111/pce.12021 23050986

[B7] Baker N. R. Harbinson J. Kramer D. M. (2007). Determining the limitations and regulation of photosynthetic energy transduction in leaves. *Plant Cell Environ.* 30 1107–1125. 10.1111/j.1365-3040.2007.01680.x 17661750

[B8] Barrs H. Weatherley P. (1962). A Re-examination of the relative turgidity technique for estimating water deficits in leaves. *Austr. J. Biol. Sci.* 15:413. 10.1071/BI9620413

[B9] Blum A. (2017). Osmotic adjustment is a prime drought stress adaptive engine in support of plant production. *Plant Cell Environ.* 40 4–10. 10.1111/pce.12800 27417527

[B10] Carillo P. Gibon Y. PrometheusWiki Contributors (2011). *Extraction and Determination of Proline. PrometheusWiki-Protocols in Ecological & Environmental Plant Physiology.* Available online at: prometheuswiki.org/tiki-custom_home.php (accessed October 10, 2016).

[B11] Cechin I. Rossi S. C. Oliveira V. C. Fumis T. F. (2006). Photosynthetic responses and proline content of mature and young leaves of sunflower plants under water deficit. *Photosynthetica* 44 :143. 10.1007/s11099-005-0171-2

[B12] Chaves M. M. Maroco J. P. Pereira J. S. (2003). Understanding plant responses to drought — from genes to the whole plant. *Funct. Plant Biol.* 30 239–264. 10.1071/fp02076 32689007

[B13] Chaves M. M. Oliveira M. M. (2004). Mechanisms underlying plant resilience to water deficits: prospects for water-saving agriculture. *J. Exp. Bot.* 55 2365–2384. 15475377 10.1093/jxb/erh269

[B14] de Campos M. K. F. de Carvalho K. de Souza F. S. Marur C. J. Pereira L. F. P. Filho J. C. B. (2011). Drought tolerance and antioxidant enzymatic activity in transgenic ‘Swingle’ citrumelo plants over-accumulating proline. *Environ. Exp. Bot.* 72 242–250. 10.1016/j.envexpbot.2011.03.009

[B15] Dien D. C. Mochizuki T. Yamakawa T. (2019). Effect of various drought stresses and subsequent recovery on proline, total soluble sugar and starch metabolisms in Rice (*Oryza sativa* L.) varieties. *Plant Product. Sci.* 22 530–545. 10.1080/1343943X.2019.1647787

[B16] Dutra de Souza J. de Andrade Silva E. M. Coelho Filho M. A. Morillon R. Bonatto D. Micheli F. (2017). Different adaptation strategies of two citrus scion/rootstock combinations in response to drought stress. *PLoS One* 12:177993. 10.1371/journal.pone.0177993 28545114 PMC5435350

[B17] FAOSTAT (2019). Available online at: from http://www.fao.org/faostat/en/#data/QC/ (accessed July 23, 2020).

[B18] Farooq M. Hussain M. Wahid A. Siddique K. H. M. (2012). “Drought stress in plants: an overview,” in *Plant Responses to Drought Stress: From Morphological to Molecular Features*, ed. Aroca R. (Berlin: Springer), 1–33. 10.1007/978-3-642-32653-0_1

[B19] Farooq M. Wahid A. Kobayashi N. Fujita D. Basra S. M. A. (2009). Plant drought stress: effects, mechanisms and management. *Agron. Sustain. Dev.* 29 185–212. 10.1051/agro:2008021

[B20] Flexas J. Badger M. Chow W. S. Medrano H. Osmond C. B. (1999). Analysis of the relative increase in photosynthetic O2 uptake when photosynthesis in grapevine leaves is inhibited following low night temperatures and/or water stress. *Plant Physiol.* 121 675–684. 10.1104/pp.121.2.675 10517860 PMC59431

[B21] Flexas J. Bota J. Loreto F. Cornic G. Sharkey T. D. (2004). Diffusive and metabolic limitations to photosynthesis under drought and salinity in C(3) plants. *Plant Biol. (Stutt. Germ.)* 6 269–279. 10.1055/s-2004-820867 15143435

[B22] Flexas J. Carriquí M. Nadal M. (2018). Gas exchange and hydraulics during drought in crops: who drives whom? *J. Exp. Bot.* 69 3791–3795. 10.1093/jxb/ery235 30032258 PMC6054177

[B23] Foyer C. H. Ruban A. V. Noctor G. (2017). Viewing oxidative stress through the lens of oxidative signalling rather than damage. *Biochem. J.* 474 877–883. 10.1042/BCJ20160814 28270560 PMC5469280

[B24] Gallé A. Haldimann P. Feller U. (2007). Photosynthetic performance and water relations in young pubescent oak (*Quercus pubescens*) trees during drought stress and recovery. *New Phytol.* 174 799–810. 10.1111/j.1469-8137.2007.02047.x 17504463

[B25] Gonçalves L. P. Alves T. F. O. Martins C. P. S. de Sousa A. O. dos Santos I. C. Pirovani C. P. (2016). Rootstock-induced physiological and biochemical mechanisms of drought tolerance in sweet orange. *Acta Physiol. Plant.* 38:174. 10.1007/s11738-016-2198-3

[B26] Grosser J. W. Barthe G. A. Castle B. Gmitter F. G. Lee O. Jr. (2015). The development of improved tetraploid citrus rootstocks to facilitate advanced production systems and sustainable citriculture in Florida. *Acta Horticult.* 1065 319–327. 10.17660/ActaHortic.2015.1065.38

[B27] Hodges D. M. DeLong J. M. Forney C. F. Prange R. K. (1999). Improving the thiobarbituric acid-reactive-substances assay for estimating lipid peroxidation in plant tissues containing anthocyanin and other interfering compounds. *Planta* 207 604–611. 10.1007/s004250050524 28456836

[B28] Jiang Z. Y. Woollard A. C. Wolff S. P. (1991). Lipid hydroperoxide measurement by oxidation of Fe2+ in the presence of xylenol orange. Comparison with the TBA assay and an iodometric method. *Lipids* 26 853–856. 1795606 10.1007/BF02536169

[B29] Jones K. D. Reed S. M. Rinehart T. A. (2007). Analysis of ploidy level and its effects on guard cell length, pollen diameter, and fertility in *Hydrangea macrophylla*. *HortScience* 42 483–488. 10.21273/HORTSCI.42.3.483

[B30] Khalid M. F. Hussain S. Anjum M. A. Ahmad S. Ali M. A. Ejaz S. (2020). Better salinity tolerance in tetraploid vs diploid volkamer lemon seedlings is associated with robust antioxidant and osmotic adjustment mechanisms. *J. Plant Physiol.* 244:153071. 10.1016/j.jplph.2019.153071 31756571

[B31] Krall J. P. Edwards G. E. (1992). Relationship between photosystem II activity and CO2 fixation in leaves. *Physiol. Plant.* 86 180–187. 10.1111/j.1399-3054.1992.tb01328.x

[B32] Lawlor D. W. Cornic G. (2002). Photosynthetic carbon assimilation and associated metabolism in relation to water deficits in higher plants. *Plant Cell Environ.* 25 275–294. 10.1046/j.0016-8025.2001.00814.x 11841670

[B33] Lelieveld J. Hadjinicolaou P. Kostopoulou E. Chenoweth J. El Maayar M. Giannakopoulos C. (2012). Climate change and impacts in the Eastern Mediterranean and the Middle East. *Clim. Change* 114 667–687. 10.1007/s10584-012-0418-4 25834296 PMC4372776

[B34] Liao T. Wang Y. Xu C. P. Li Y. Kang X. Y. (2018). Adaptive photosynthetic and physiological responses to drought and rewatering in triploid Populus populations. *Photosynthetica* 56 578–590. 10.1007/s11099-017-0704-5

[B35] Lourkisti R. Froelicher Y. Herbette S. Morillon R. Tomi F. Gibernau M. (2020). Triploid citrus genotypes have a better tolerance to natural chilling conditions of photosynthetic capacities and specific leaf volatile organic compounds. *Front. Plant Sci.* 11:330. 10.3389/fpls.2020.00330 32391024 PMC7189121

[B36] Luna C. Garcia−Seffino L. Arias C. Taleisnik E. (2000). Oxidative stress indicators as selection tools for salt tolerance. *Plant Breed.* 119 341–345. 10.1046/j.1439-0523.2000.00504.x

[B37] Machado D. F. S. P. Machado E. C. Machado R. S. Ribeiro R. V. (2010). Effects of low night temperature and rootstocks on diurnal variation of leaf gas exchange rates and photochemical activity of “Valência” sweet orange plants. *Rev. Brasil. Fruticult.* 32 351–359. 10.1590/S0100-29452010005000064

[B38] Maxwell K. Johnson G. N. (2000). Chlorophyll fluorescence–a practical guide. *J. Exp. Bot.* 51 659–668. 10938857 10.1093/jxb/51.345.659

[B39] Medina C. L. Souza R. P. Machado E. C. Ribeiro R. V. Silva J. A. B. (2002). Photosynthetic response of citrus grown under reflective aluminized polypropylene shading nets. *Sci. Horticult.* 96 115–125. 10.1016/S0304-4238(02)00085-7

[B40] Merilo E. Yarmolinsky D. Jalakas P. Parik H. Tulva I. Rasulov B. (2018). Stomatal VPD response: there is more to the story than ABA. *Plant Physiol.* 176 851–864. 10.1104/pp.17.00912 28986421 PMC5761775

[B41] Miranda M. T. Da Silva S. F. Silveira N. M. Pereira L. Machado E. C. Ribeiro R. V. (2020). Root osmotic adjustment and stomatal control of leaf gas exchange are dependent on citrus rootstocks under water deficit. *J. Plant Growth Regul.* 2 1–9. 10.1007/s00344-020-10069-5

[B42] Mittler R. (2002). Oxidative stress, antioxidants and stress tolerance. *Trends Plant Sci.* 7 405–410. 12234732 10.1016/s1360-1385(02)02312-9

[B43] Navarro L. Aleza P. Cuenca J. Juárez J. José A. Pina C. O. (2015). The mandarin triploid breeding program in Spain. *Acta Horticult.* 1065 389–395. 10.17660/ActaHortic.2015.1065.48

[B44] Niinemets U. Díaz-Espejo A. Flexas J. Galmés J. Warren C. R. (2009). Importance of mesophyll diffusion conductance in estimation of plant photosynthesis in the field. *J. Exp. Bot.* 60 2271–2282. 10.1093/jxb/erp063 19305021

[B45] Noctor G. Foyer C. H. (2016). Intracellular redox compartmentation and ROS-related communication in regulation and signaling. *Plant Physiol.* 171:1581. 10.1104/pp.16.00346 27208308 PMC4936564

[B46] Oliveira T. M. Yahmed J. B. Dutra J. Maserti B. E. Talon M. Navarro L. (2017). Better tolerance to water deficit in doubled diploid ‘Carrizo citrange’ compared to diploid seedlings is associated with more limited water consumption. *Acta Physiol. Plant.* 39:204. 10.1007/s11738-017-2497-3

[B47] Ollitrault P. Dambier D. Luro F. Froelicher Y. (2008). “Ploidy manipulation for breeding seedless triploid citrus,” in *Plant Breeding Reviews*, ed. Janick J. (Hoboken, NJ: John Wiley & Sons, Ltd), 323–352. 10.1002/9780470380130.ch7

[B48] Oustric J. Morillon R. Luro F. Herbette S. Lourkisti R. Giannettini J. (2017). Tetraploid Carrizo citrange rootstock (*Citrus sinensis* Osb.×*Poncirus trifoliata* L. Raf.) enhances natural chilling stress tolerance of common clementine (*Citrus clementina* Hort. ex Tan). *J. Plant Physiol.* 214 108–115. 10.1016/j.jplph.2017.04.014 28478318

[B49] Oustric J. Morillon R. Luro F. Herbette S. Martin P. Giannettini J. (2019). Nutrient deficiency tolerance in citrus is dependent on genotype or ploidy level. *Front. Plant Sci.* 10:127. 10.3389/fpls.2019.00127 30853962 PMC6396732

[B50] Padoan D. Mossad A. Chiancone B. Germana M. A. Khan P. S. S. V. (2013). Ploidy levels in Citrus clementine affects leaf morphology, stomatal density and water content. *Theor. Exp. Plant Physiol.* 25 283–290. 10.1590/S2197-00252013000400006

[B51] Pei Z. M. Murata Y. Benning G. Thomine S. Klüsener B. Allen G. J. (2000). Calcium channels activated by hydrogen peroxide mediate abscisic acid signalling in guard cells. *Nature* 406 731–734. 10.1038/35021067 10963598

[B52] Pérez-Clemente R. M. Montoliu A. Zandalinas S. I. de Ollas C. Gómez-Cadenas A. (2012). Carrizo citrange plants do not require the presence of roots to modulate the response to osmotic stress. *Sci. World J.* 2012:795396. 10.1100/2012/795396 22919353 PMC3417192

[B53] Poggi I. Polidori J. J. Gandoin J. M. Paolacci V. Battini M. Albertini M. (2007). Stomatal regulation and xylem cavitation in Clementine (Citrus clementina Hort) under drought conditions. *J. Horticult. Sci. Biotechnol.* 82 845–848. 10.1080/14620316.2007.11512316

[B54] Recupero G. R. Russo G. Recupero S. (2005). New promising citrus triploid hybrids selected from crosses between monoembryonic diploid female and tetraploid male parents. *HortScience* 40 516–520. 10.21273/HORTSCI.40.3.516

[B55] Ribeiro R. V. Machado E. C. Santos M. G. Oliveira R. F. (2009). Seasonal and diurnal changes in photosynthetic limitation of young sweet orange trees. *Environ. Exp. Bot.* 66 203–211. 10.1016/j.envexpbot.2009.03.011

[B56] Rouiss H. Cuenca J. Navarro L. Ollitrault P. Aleza P. (2017). Tetraploid citrus progenies arising from FDR and SDR unreduced pollen in 4x X 2x hybridizations. *Tree Genet. Genom.* 13 :10. 10.1007/s11295-016-1094-8

[B57] Salehi-Lisar S. Y. Bakhshayeshan-Agdam H. (2016). “Drought stress in plants: causes, consequences, and tolerance,” in *Drought Stress Tolerance in Plants: Physiology and Biochemistry*, Vol. 1 eds Hossain M. A. Wani S. H. Bhattacharjee S. Burritt D. J. Tran L.-S. P. (Manhattan, NY: Springer International Publishing), 1–16. 10.1007/978-3-319-28899-4_1

[B58] Santana-Vieira D. D. S. Freschi L. Almeida L. A. da H. Moraes D. H. S. de Neves D. M. (2016). Survival strategies of citrus rootstocks subjected to drought. *Sci. Rep.* 6:38775. 10.1038/srep38775 27996018 PMC5171762

[B59] Santini J. Giannettini J. Pailly O. Herbette S. Ollitrault P. Berti L. (2013). Comparison of photosynthesis and antioxidant performance of several Citrus and Fortunella species (Rutaceae) under natural chilling stress. *Trees* 27 71–83. 10.1007/s00468-012-0769-5

[B60] Serraj R. Sinclair T. R. (2002). Osmolyte accumulation: can it really help increase crop yield under drought conditions? *Plant Cell Environ.* 25 333–341. 11841674 10.1046/j.1365-3040.2002.00754.x

[B61] Silva M. de A. Jifon J. L. Santos C. M. dos Jadoski C. J. da Silva J. A. G. (2013). Photosynthetic capacity and water use efficiency in sugarcane genotypes subject to water deficit during early growth phase. *Braz. Arch. Biol. Technol.* 56 735–748. 10.1590/S1516-89132013000500004

[B62] Smirnoff N. (2018). Ascorbic acid metabolism and functions: a comparison of plants and mammals. *Free Radic. Biol. Med.* 122 116–129. 29567393 10.1016/j.freeradbiomed.2018.03.033 PMC6191929

[B63] Stevens R. Page D. Gouble B. Garchery C. Zamir D. Causse M. (2008). Tomato fruit ascorbic acid content is linked with monodehydroascorbate reductase activity and tolerance to chilling stress. *Plant Cell Environ.* 31 1086–1096. 10.1111/j.1365-3040.2008.01824.x 18433441

[B64] Szabados L. Savouré A. (2010). Proline: a multifunctional amino acid. *Trends Plant Sci.* 15 89–97. 10.1016/j.tplants.2009.11.009 20036181

[B65] Tabassum T. Farooq M. Ahmad R. Zohaib A. Wahid A. (2017). Seed priming and transgenerational drought memory improves tolerance against salt stress in bread wheat. *Plant Physiol. Biochem.* 118 362–369. 28711787 10.1016/j.plaphy.2017.07.007

[B66] Tan F.-Q. Tu H. Liang W.-J. Long J.-M. Wu X.-M. Zhang H.-Y. (2015). Comparative metabolic and transcriptional analysis of a doubled diploid and its diploid citrus rootstock (C. junos cv. Ziyang xiangcheng) suggests its potential value for stress resistance improvement. *BMC Plant Biol.* 15:89. 10.1186/s12870-015-0450-4 25848687 PMC4374211

[B67] Tan F.-Q. Tu H. Wang R. Wu X.-M. Xie K.-D. Chen J.-J. (2017). Metabolic adaptation following genome doubling in citrus doubled diploids revealed by non-targeted metabolomics. *Metabolomics* 13:143. 10.1007/s11306-017-1276-x

[B68] Tan F.-Q. Zhang M. Xie K.-D. Fan Y.-J. Song X. Wang R. (2019). Polyploidy remodels fruit metabolism by modifying carbon source utilization and metabolic flux in Ponkan mandarin (*Citrus reticulata* Blanco). *Plant Sci.* 289:110276. 10.1016/j.plantsci.2019.110276 31623787

[B69] Wang X. Cheng Z.-M. Zhi S. Xu F. (2016). Breeding triploid plants: a review. *Czech J. Genet. Plant Breed.* 52 41–54. 10.17221/151/2015-CJGPB

[B70] Wang X. Mao Z. Zhang J. Hemat M. Huang M. Cai J. (2019). Osmolyte accumulation plays important roles in the drought priming induced tolerance to post-anthesis drought stress in winter wheat (*Triticum aestivum* L.). *Environ. Exp. Bot.* 166:103804. 10.1016/j.envexpbot.2019.103804

[B71] Wei T. Wang Y. Xie Z. Guo D. Chen C. Fan Q. (2019). Enhanced ROS scavenging and sugar accumulation contribute to drought tolerance of naturally occurring autotetraploids in Poncirus trifoliata. *Plant Biotechnol. J.* 17 1394–1407. 10.1111/pbi.13064 30578709 PMC6576089

[B72] Zhu J.-K. (2002). Salt and drought stress signal transduction in plants. *Ann. Rev. Plant Biol.* 53 247–273. 10.1146/annurev.arplant.53.091401.143329 12221975 PMC3128348

