# Supplementary material for: Triploidy in Citrus Genotypes Improves Leaf Gas Exchange and Antioxidant Recovery From Water Deficit
Source: Front Plant Sci. 2021 Feb 19;11:615335. doi: 10.3389/fpls.2020.615335 (PMC7933528; doi:10.3389/fpls.2020.615335)
Supplement: Supplementary file 1 [file Data_Sheet_1.docx]

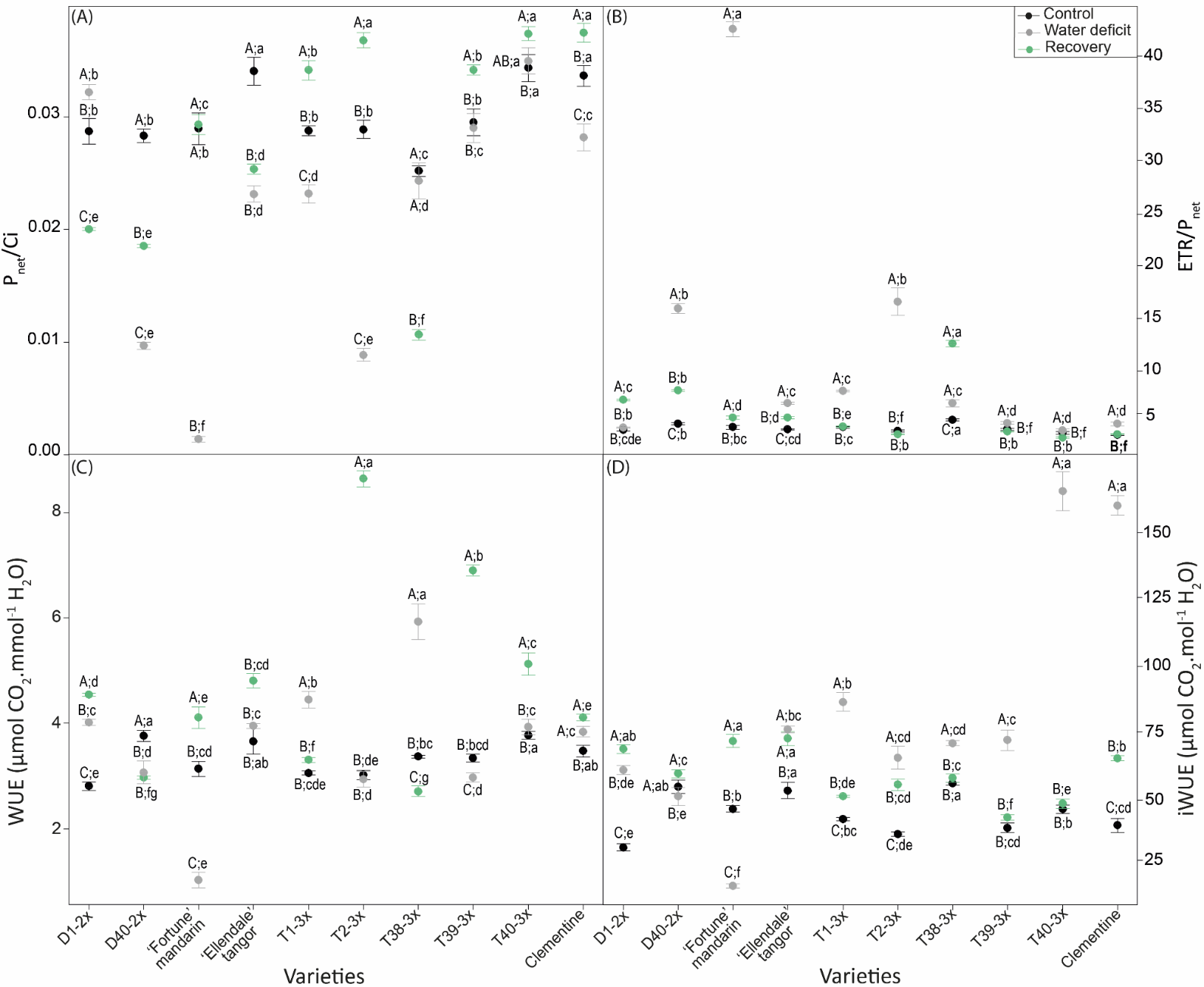


**Figure 1:** Changes in (A) carboxylation efficiency (*P_net_/Ci*), (B) ETR/*P_net_* ratio, (C) instantaneous (WUE) and (D) intrinsic (iWUE) water use efficiencies in varieties under three different water conditions: control (black points), water deficit (grey points) and after re-watering (recovery; green points). All data are mean values (±S.E.) of 15 independent measurements (*n*=15). Data were analysed using ANOVA and Fisher LSD test (P<0.05). Different capital letters indicate significant changes between the conditions (control, water deficit, recovery) for each variety while different lower case letters indicate changes between the varieties for each condition.


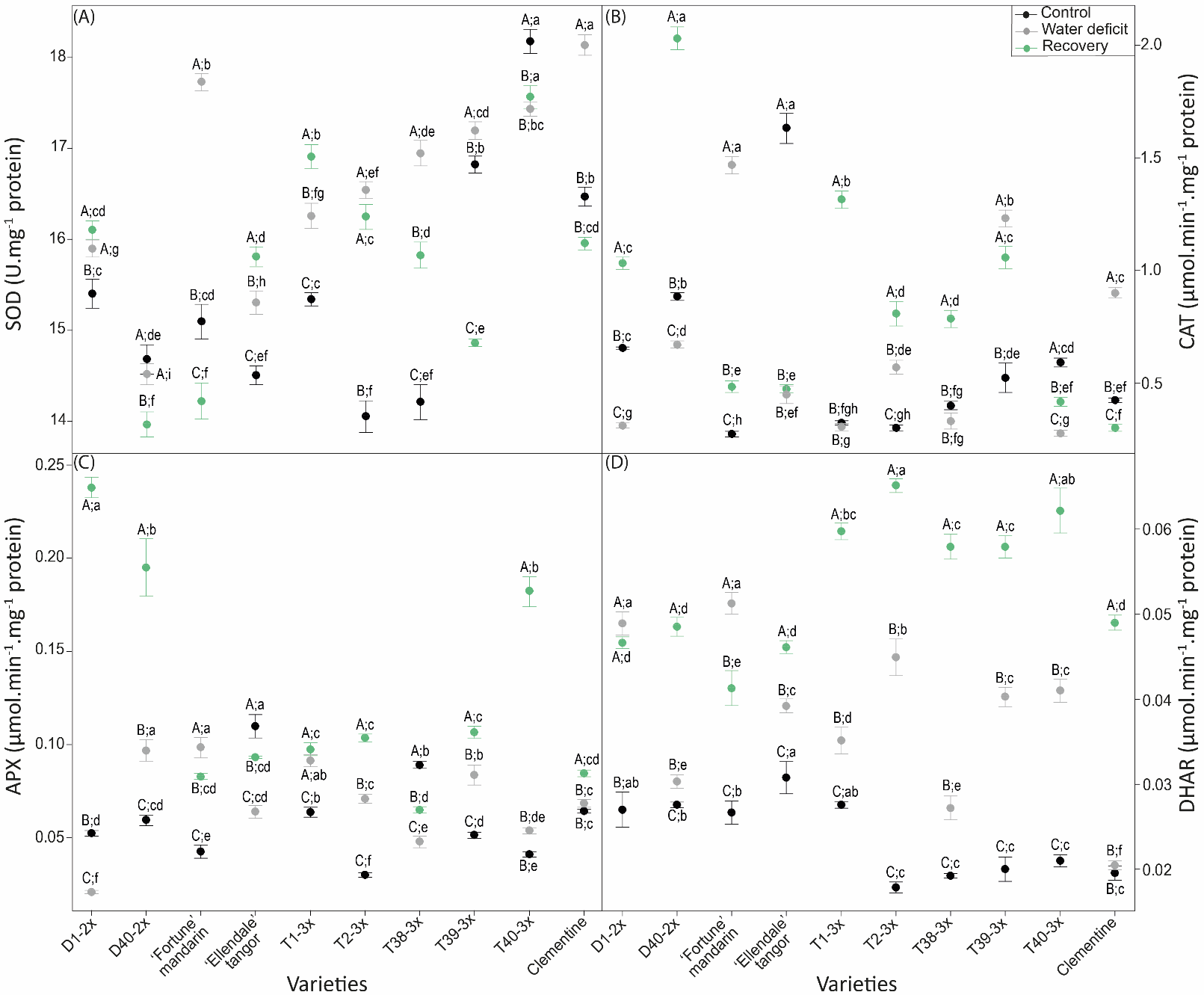


**Figure 2:** Evolution of enzymatic activities of (A) superoxide dismutase (SOD), (B) catalase (CAT), (C) ascorbate peroxidase (APX) and dehydroascorbate reductase (DHAR) in leaves of varieties under three different water conditions: control (black points), water deficit (grey points) and after rehydration (recovery; green points). All data are mean values (±S.E.) of three independent measurements (*n*=3). Data were analysed using ANOVA and Fisher LSD test (P<0.05). Different capital letters indicate significant changes between the conditions (control, water deficit, recovery) for each variety while different lower case letters indicate changes between the varieties for each condition.


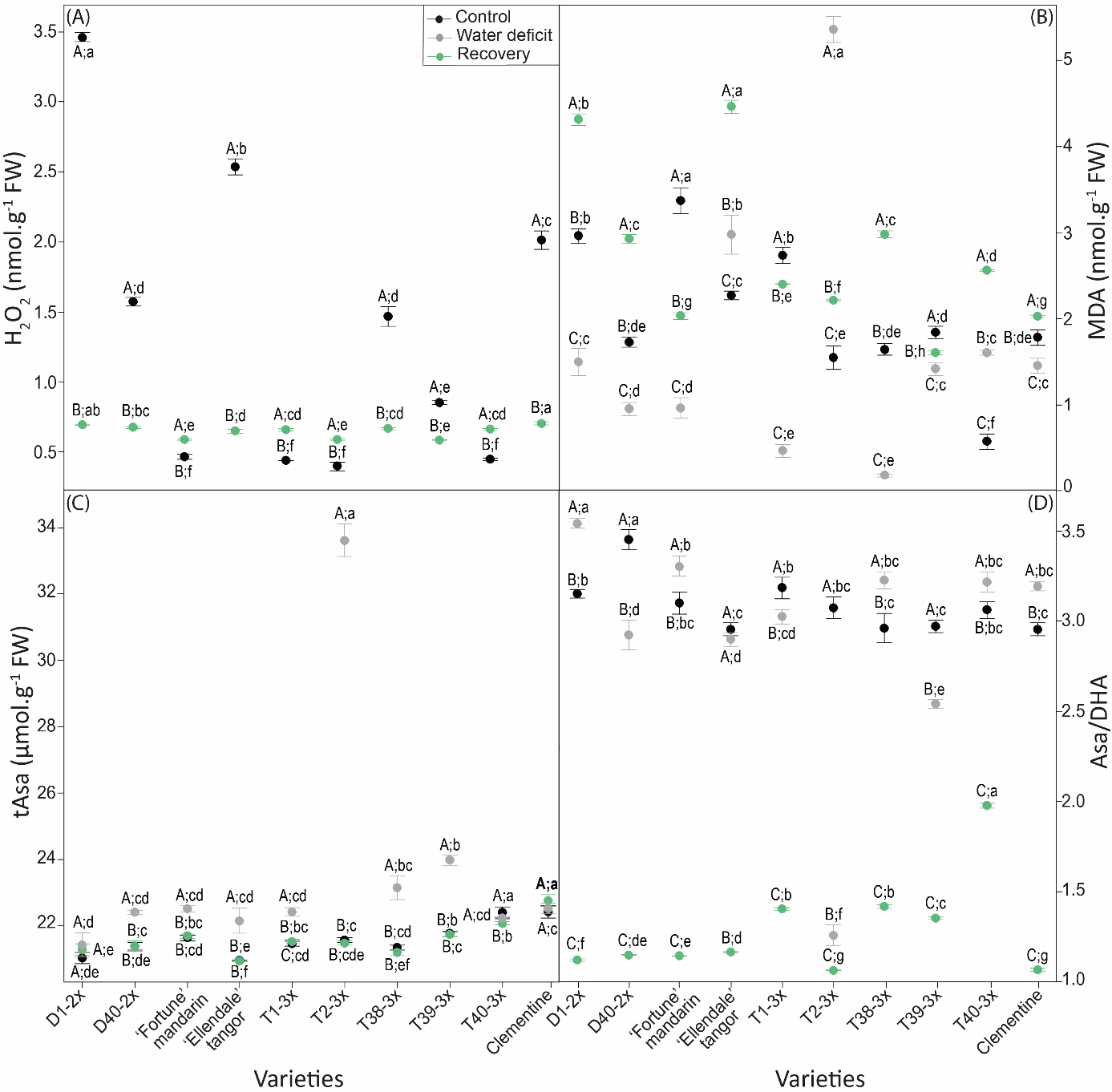


**Figure 3:** Evolution of oxidative markers (A: H_2_O_2_; B: MDA) and antioxidants (C: tAsa; D: Asa/DHA) in roots of varieties under three different water conditions: control (black points), water deficit (grey points) and after rehydration (recovery; green points). All data are mean values (±S.E.) of three independent measurements (*n*=3). Data were analysed using ANOVA and Fisher LSD test (P<0.05). Different capital letters indicate significant changes between the conditions (control, water deficit, recovery) for each variety while different lower case letters indicate changes between the varieties for each condition.


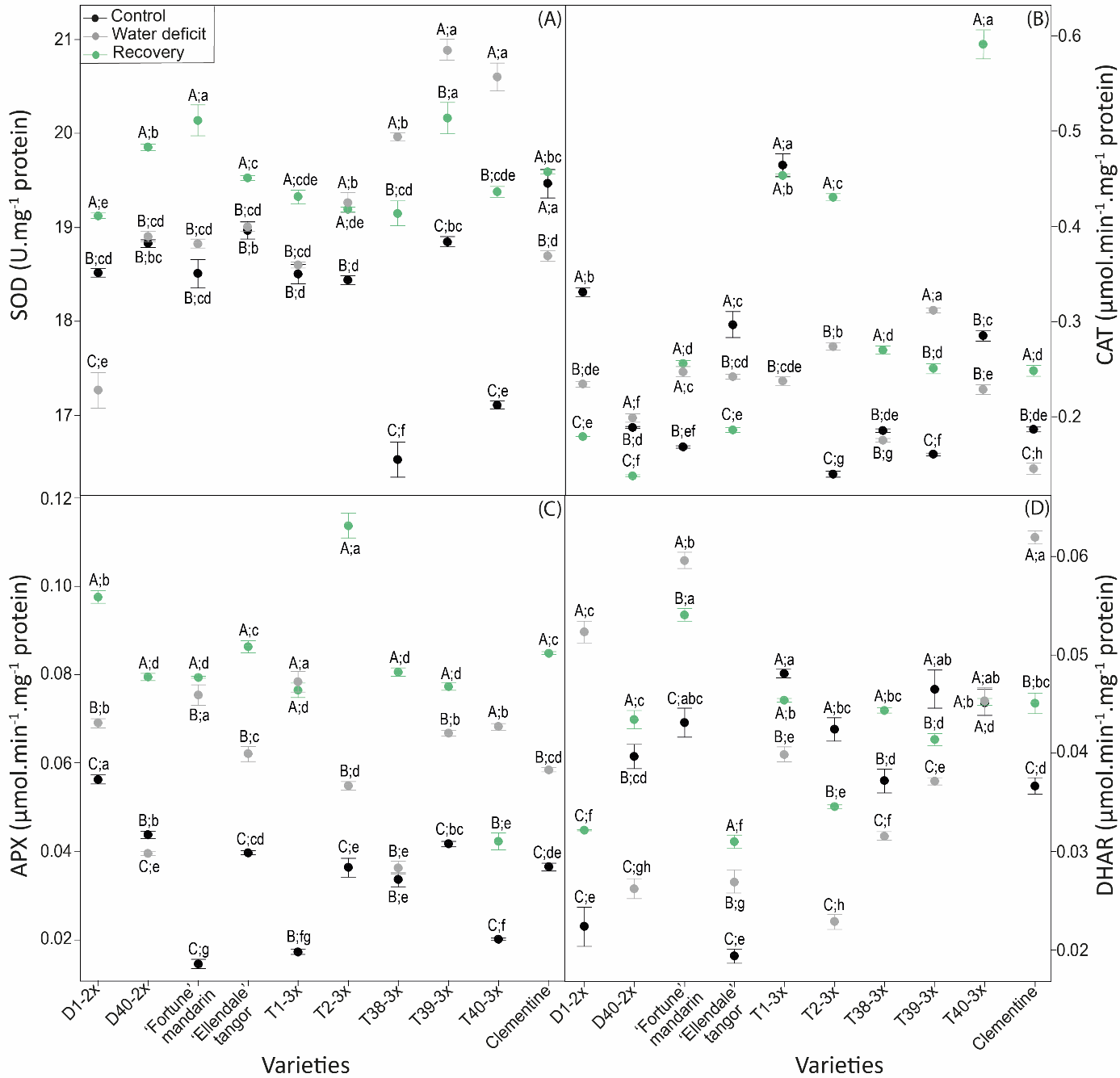


**Figure 4:** Evolution of enzymatic antioxidant activities of (A) SOD, (B) CAT, (C) APX and (D) DHAR in roots of varieties under three different water conditions: control (black points), water deficit (grey points) and after rehydration (recovery; green points). All data are mean values (±S.E.) of three independent measurements (*n*=3). Data were analysed using ANOVA and Fisher LSD test (P<0.05). Different capital letters indicate significant changes between the conditions (control, water deficit, recovery) for each variety while different lower case letters indicate changes between the varieties for each condition.
